# The contribution of breastfeeding to the prevention of breast cancer

**DOI:** 10.18332/ejm/114885

**Published:** 2019-11-29

**Authors:** Sofia Dimou, Asimenia Mamakou, Evanthia Konstantaki, Magdaleni Spanou

**Affiliations:** 1 General, Obstetrics and Pediatric Clinic Mitera, Athens, Greece

**Keywords:** breast cancer, breastfeeding, mutation, BRCA1, BRCA2

**Dear Editor,**

Breastfeeding and its duration appear to have a protective effect against the risk of breast cancer, especially for women with mutations in predominantly the BRCA1 gene but also the BRCA2 gene. The interaction between breastfeeding and cancer is an area of significant importance that should be highlighted when engaging during antenatal care.

Specifically, Giudici et al.^1^ have shown that if women in developed countries were breastfeeding six months longer there would be a 5% reduction in the incidence of breast cancer every year. However, if they had been breastfeeding for more than 12 months, the reduction would be 11%. Similarly, Jernstrom et al.^2^ found that women with a mutation in the BRCA1 gene, who were breastfeeding their babies for over one year, were 45% less susceptible to breast cancer compared to those who did not breastfeed. In contrast, they found no correlation between maternal breastfeeding and women with mutations in the BRCA2 gene.

Research by De Silva et al.^3^ in a Sri Lanka population suggested that an average of 12 months or more of breastfeeding reduces the risk of breast cancer. In particular, they found that for a breastfeeding duration of 12 to 23 months, the risk of developing breast cancer decreased, as supported by the study of Awatef et al.^4^ that noted a significantly reduced risk of breast cancer for women who had 2 to 3 children and a breastfeeding duration of 72 months.

In another study, by Kotsopoulos et al.^5^, it was found that breastfeeding protects against the incidence of breast cancer in women with the BRCA1 gene mutation. In contrast, maternal breastfeeding is not protective of women with BRCA2 gene mutation, possibly due to different mechanisms of carcinogenesis.

Furthermore, Giudici et al.^1^ found that breastfeeding reduces the likelihood of another Luminal B invasive breast cancer in premenopausal White women for every 12 months of breastfeeding. In contrast, it does not appear to reduce the likelihood of developing a milder type of breast cancer such as Luminal A.

Results of research by Jeong et al.^6^ in a Korean population have shown that the combination of an increase in the breastfeeding period to over 13 months and having at least two children leads to a reduction in the risk of breast cancer by 50%. Additionally, women who had been breastfeeding for more than 25 months had a reduced risk of breast cancer by 56%, regardless of their menopausal status. Finally, even women having only one child may have a reduced risk of developing breast cancer after breastfeeding.

The benefits of breastfeeding for mothers, infants, the economy and the environment are of unparalleled value. Hence, it is essential in midwifery practice to further raise awareness among health professionals, women and young mothers regarding the advantages of breastfeeding, so that more mothers and infants enjoy the benefits of breastfeeding, which may also provide long-term protection against breast cancer.
